# Supplementary material for: Increased labor losses and decreased adaptation potential in a warmer world
Source: Nat Commun. 2021 Dec 14;12:7286. doi: 10.1038/s41467-021-27328-y (PMC8671389; doi:10.1038/s41467-021-27328-y)
Supplement: Supplementary file 1 — Editor Summary [file 41467_2021_27328_MOESM1_ESM.docx]

Outdoor workers may need to adapt to warming by moving labor from midday to cooler hours. Here the authors find this adaptation strategy loses efficacy under additional climate change due to increased heat exposure in the coolest hours of the day.
