# Supplementary material for: Increased labor losses and decreased adaptation potential in a warmer world
Source: Nat Commun. 2021 Dec 14;12:7286. doi: 10.1038/s41467-021-27328-y (PMC8671389; doi:10.1038/s41467-021-27328-y)
Supplement: Supplementary file 2 — Supplementary Information [file 41467_2021_27328_MOESM2_ESM.pdf]

Supplementary Information for

Increased labor losses and decreased adaptation potential in a warmer world

L. A. Parsons[1], D. Shindell[1], and M. Tigchelaar[2], Y. Zhang[1,3] J. Spector [4]

[1]Nicholas School of the Environment, Duke University, Durham, NC, USA
[2]Center for Ocean Solutions, Stanford University, Stanford, CA, USA
[3]Department of Environmental Sciences and Engineering, University of North Carolina at Chapel Hill, Chapel Hill, NC, USA
[4] Departments of Environmental and Occupational Health Sciences and Medicine, University of Washington, Seattle, WA, USA

This file contains

Supplementary Text 1
Supplementary Figures 1-15
Supplementary Tables 1-2

Supplementary Text 1.

Differences in warming patterns: daily mean, maximum, minimum temperatures

Daily mean, maximum, and minimum temperatures over tropical land areas warm at a rate of ~1-1.2°C per degree of global warming (Supplementary Figure 4). We are interested in heat exposure at the hottest and coolest hours of the day, so we also examine changes in diurnal temperature range[1] (DTR) per degree of warming by calculating the differences in daily maximum minus minimum warming patterns. Across much of the global land area, there is little agreement among CMIP6 models in the average change in the DTR. However, in parts of the American tropics, southern Europe, and Africa, these models agree on faster warming in daytime maximum relative to minimum temperatures. These models also tend to agree that minimum temperatures will rise more quickly than maximum temperatures in the southern United States and Southwest Asia. Nonetheless, we rely on simulated changes in daily mean temperatures and humidity to calculate future changes in sWBGT because observed and projected changes in DTR are expected to be small relative to changes in the mean[2-4]. We have excluded the CMIP6 NorESM2-LM model from our analysis of tasmax and tasmin in the 1%CO2 experiment because these variables showed unrealistically high and low changes over land areas as compared to the higher-resolution NorESM2-MM and the other CMIP6 models.

Estimating economic productivity impacts of heavy labor productivity losses

To estimate the country-level economic impacts of lost work productivity due to heat exposure, we multiply the work hour loss in each of our two heavy labor sectors (agriculture, forestry and fisheries; construction) by the average hourly value added per worker in each sector. For each country, we calculate average value added per worker in agriculture, forestry and fisheries by dividing total value added in 2017 (in current local currency units, LCU; World Bank (WB) data product ID: NV.AGR.TOTL.CN) in agriculture, forestry and fisheries by total, working-age employment in agriculture, forestry and fisheries, then dividing by the number of hours worked in a year used in the main text (12*365). The World Bank does not report value added in construction, so we instead use the value added data for "industry (incl. construction)", and calculate average worker productivity in each country by dividing total value added in 2017 in industry (including construction) (ID: NV.IND.TOTL.CN) by total employment in industry. For each country, we calculate total employment in agriculture, forestry and fisheries and in industry, respectively, as the product of total population (ID: API_SP.POP.TOTL), percent of total population ages 15-64 (ID: API_SP.POP.1564.TO.ZS), percent of employed population ages 15+ (ID: API_SL.EMP.TOTL.SP.ZS), and percent of employed population ages 15+ in agriculture, forestry and fisheries (ID: API_SL.AGR.EMPL.ZS), and in industry including construction (ID: API_SL.IND.EMPL.ZS), respectively. We convert from 2017 Local Currency Units (LCU) to 2017 purchasing power parity (PPP)-adjusted international dollars (2017 PPP$) by dividing a country's LCU by its gross domestic product (GDP) 2017 PPP conversion rate (LCU per USD). We sum value added losses over all countries (n=163) to obtain the estimated global output loss.

Sensitivity of results to use of hourly vs mean and maximum sWBGT

To estimate labor losses during the 12-hour workday, we calculate the daily mean sWBGT, daily maximum sWBGT, and the halfway point between these two values, and assume 4 hours is spent near each of these values in the 12-hour work day (4*sWBGT max + 4*sWBGT mean + 4*sWBGT half). We compared results from the '4+4+4' approximation against the losses calculated from the hourly sWBGT data (e.g., used the hourly data from the 12-hour workday as opposed to assuming 3 hours is spent at the maximum, minimum, and mean halfway point) for the cities shown in Figure 1 and for the global sums of labor lost. We found differences are <5% between the '4+4+4' approximation and the hourly data. For Atlanta the difference in work time lost is 1 minute (17 minutes for the hourly data, 16 minutes for the 4+4+4 method), for Doha the difference is 7 minutes (169 minutes for the hourly data, 176 minutes for the 4+4+4 method), and for New Delhi the difference is 3 minutes (105 minutes for the hourly data, 108 minutes for the 4+4+4 method). Global sums of labor lost similarly show <2% differences between the '4+4+4' method and the hourly data (~228 billion lost hours/year for the '4+4+4' method, and ~231 billion lost hours/year for the hourly data). These global differences among methods are much smaller than the interannual variability of hours lost/year over the 2001-2020 time period (standard deviation: ~27 billion lost hours/year).

Warming patterns in CMIP6 SSP5-8.5 vs idealized 1pctCO2 experiments

Warming patterns are generally similar in the idealized 1pctCO2 (Figure 2; Supplementary Figures 3-6) and 21st century SSP5-8.5 experiments, with differences in magnitudes of local warming patterns that are <10% (Supplementary Figure 15). However, in a few isolated northern hemisphere locations, most of the CMIP6 models (>75%) agree that the magnitude of local warming is greater in the SSP5-8.5 experiment than the 1pctCO2 experiment; many of these locations also show large projected decreases in aerosol emissions in the SSP5-8.5 experiment (not shown). Nonetheless, for most land areas there is not good agreement among CMIP6 models in the sign of difference in magnitude of change among these experiments (stippled areas show disagreement in Supplementary Figure 15), and even in the few locations where the sign is agreed upon, these differences are <10% in the multi-model median. Additionally, a sensitivity test of our future labor loss estimates using warming patterns from the SSP5-8.5 experiment shows no noticeable differences in global labor loss estimates (not shown).

21st century warming pathways in CMIP6 SSP3-7.0 and SSP5-8.5

We examine sWBGT and labor impacts of various global warming 'levels' relative to the recent past (2001-2020): 0°C (present-day), +1°C, +2°C, +3°C, and +4°C. Analysis of annual, global mean 2-m air temperature in the CMIP6 SSP3-7.0 and SSP5-8.5 experiments shows that by the end of the century, some CMIP6 models project additional warming of almost 5°C (SSP3-7.0) or 6°C (SSP5-8.5) relative to present-day, but most models fall within the 2.5-5°C range under SSP5-8.5 and the 2-4°C range in SSP3-7.0. Under SSP3-7.0, these models project an additional 1°C of warming relative to 2015-2024 mean as early as 2035 (median: 2052), 2°C as early as 2056 (median: 2074), 3°C as

early as 2073 (median: 2098), and 4°C as early as 2089 (median: after end of century; Supplementary Figure 14). Given that future greenhouse gas emissions and warming pathways are highly uncertain, here we rely on patterns of sWBGT change as the globe warms, which have been shown to be robust in global climate models[5], instead of an actual emissions pathway. Additionally, as discussed above, differences in warming patterns among the 1%CO2 simulation and the SSP5-8.5 experiment are <10% (Supplementary Figure 15).

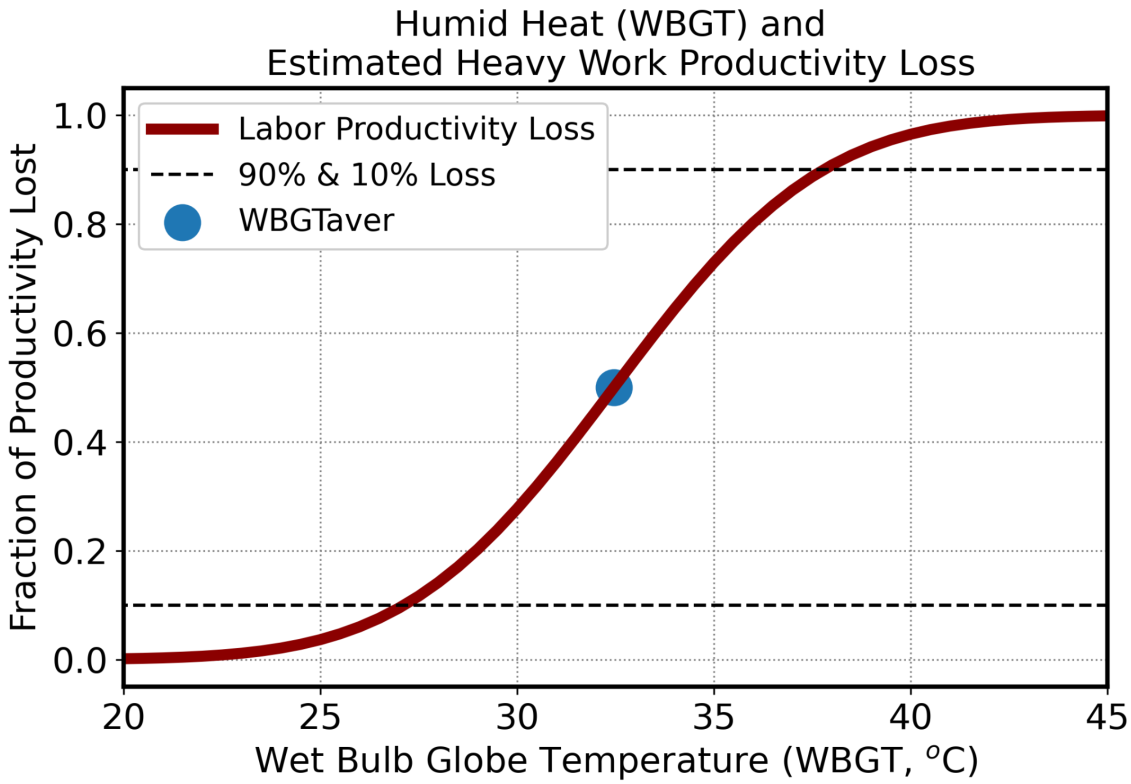

Supplementary Figure 1. Fraction of heavy labor productivity lost as a function of heat exposure (Wet Bulb Globe Temperatures, or WBGT) for outdoor workers. This ERF is based on the ERFs used in other recent global labor productivity studies[6,7]. The red line shows labor productivity loss as a function of heat exposure, the blue dot shows the 'WBGT average' value used in the equation shown in the Methods section of the main text, and the horizontal dashed lines denote 10% and 90% productivity losses.

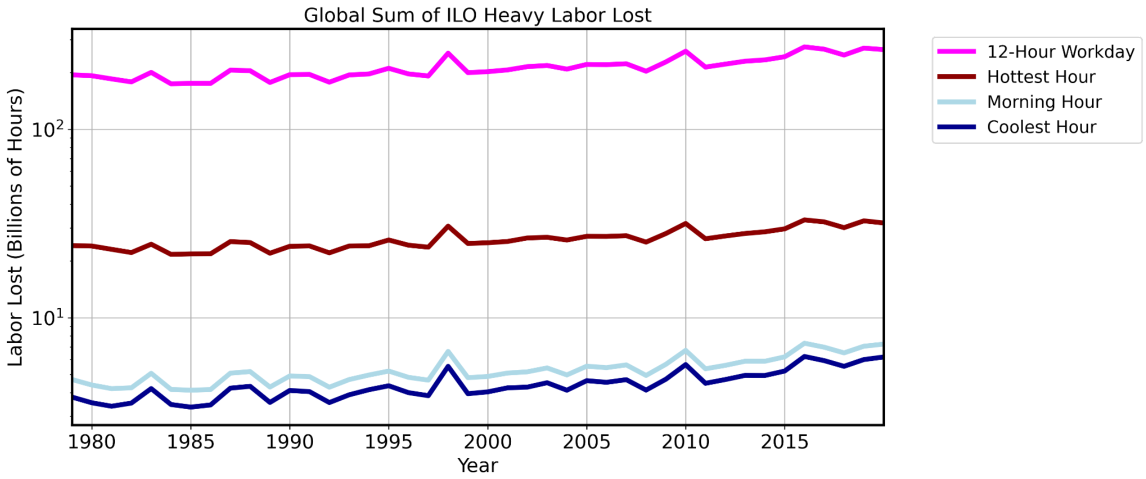

Supplementary Figure 2. Global sums of ILO heavy labor sector (agriculture, fisheries, forestry, and construction) losses 1979-2020 using ERA5 to calculate simplified Wet Bulb Globe Temperatures (sWBGT). Population and labor are held constant using GPWv4 population data for 2017 and the latest data available for ILO, with only sWBGT values varying. Note the log scale on the y-axis. ‘Morning hour’ refers to the third coolest hour of the day.

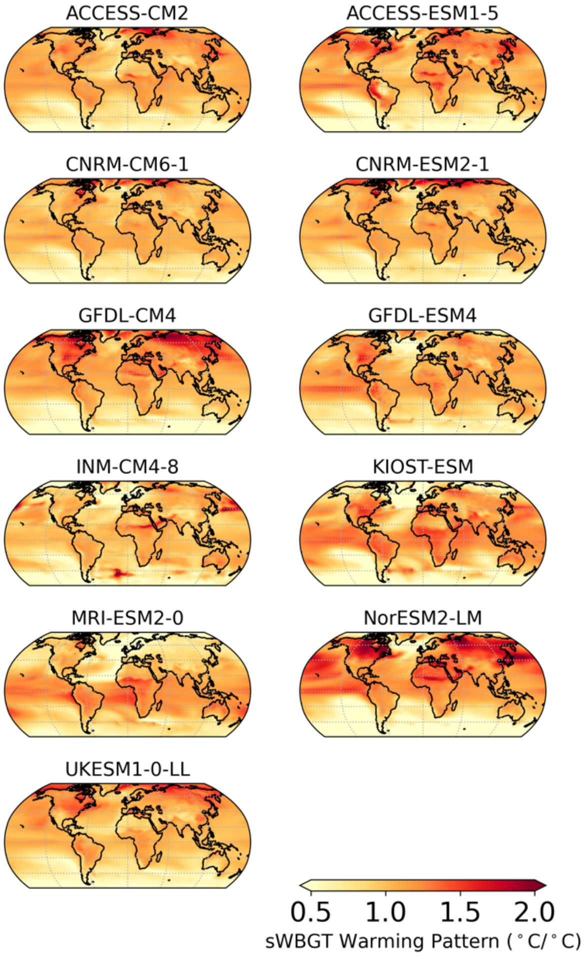

Supplementary Figure 3. Maps showing the warming patterns for the 75th percentile of daily sWBGT for the individual CMIP6 1%CO2 simulations used in the main text.

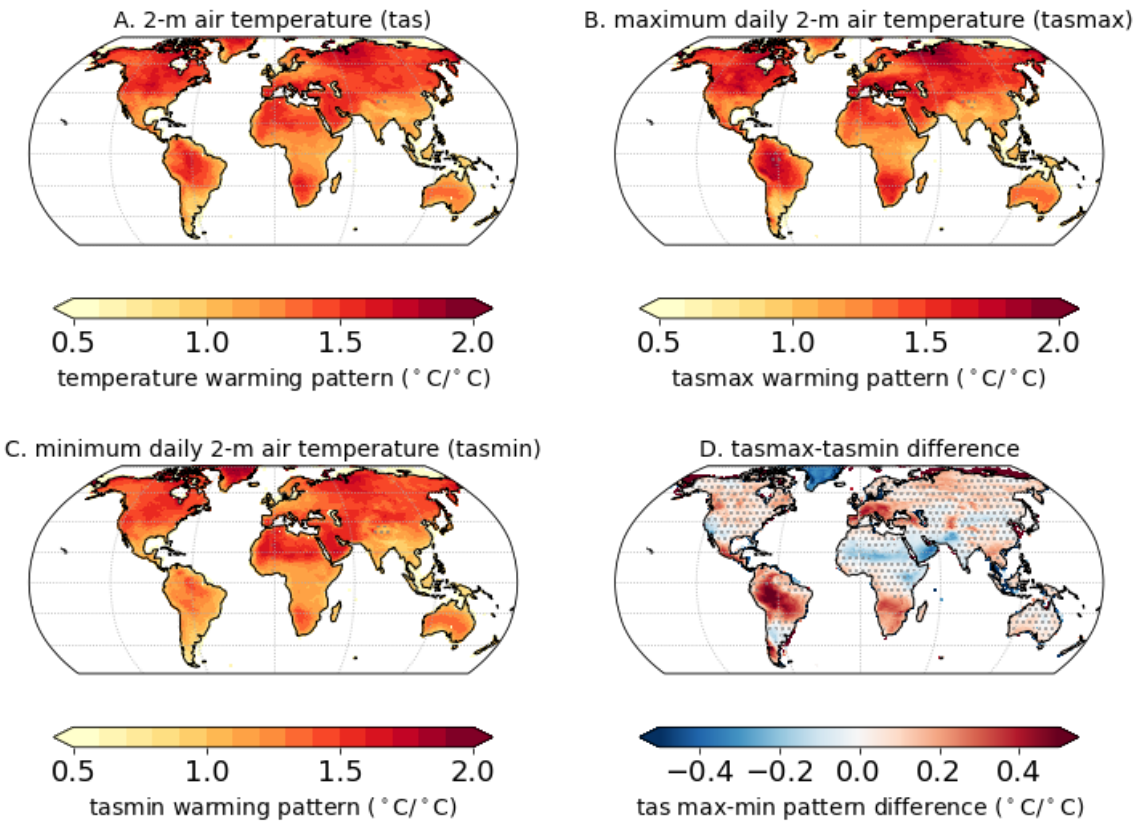

Supplementary Figure 4. Local warming patterns (local change per degree of global warming) for the annual 75th percentile of daily mean, maximum, and minimum 2-m surface air temperature (tas, tasmax, tasmin) and difference in daily maximum and minimum 2-m surface air temperature (tasmax-tasmin) in 20 Coupled Modeling Intercomparison Project, Phase 6 (CMIP6) 1%CO2 simulations. Maps show multi-model median values for each grid point, with stippling on warming pattern maps where there is disagreement in the magnitude of local change (coefficient of variation >0.35). Stippling on the tasmax-tasmin map (blue-white-red colormap) shows where <75% of CMIP6 models agree on the sign of difference in tasmax-tasmin per degree of global warming. We have excluded the CMIP6 NorESM2-LM model from our analysis of tasmax and tasmin in the 1%CO2 experiment because these variables showed unrealistically high and low changes over land areas as compared to the higher-resolution NorESM2-MM and the other CMIP6 models.

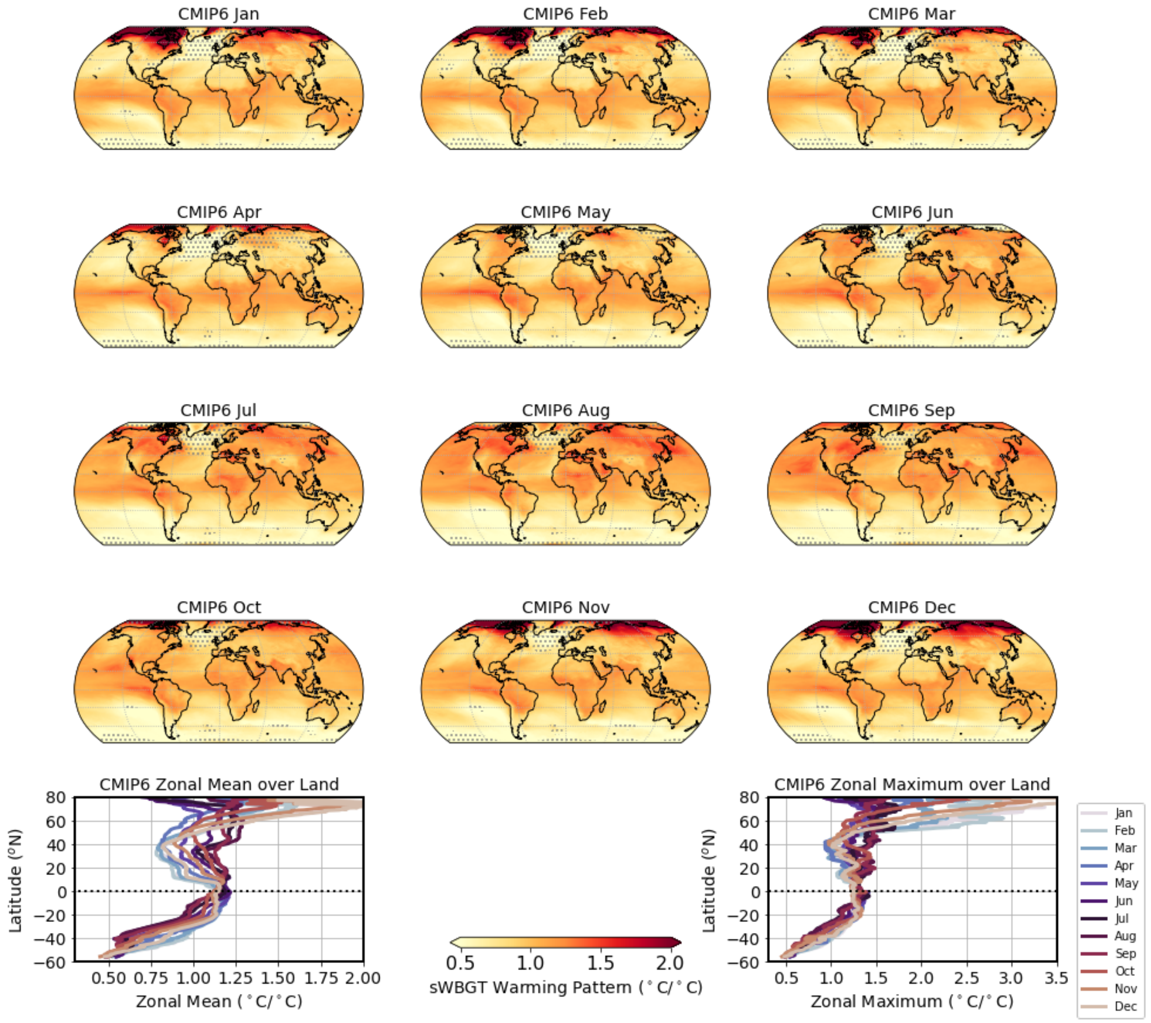

Supplementary Figure 5. Maps showing multi-model median simplified Wet Bulb Globe Temperature (sWBGT) monthly warming patterns per degree of global warming in 21 CMIP6 1%CO2 simulations. Line plots show zonal mean (lower left) and zonal maximum (bottom right) sWBGT warming patterns over land. Stippling on maps shows where models disagree on the magnitude of local warming per degree of global warming. Note the different x-axis limits in the zonal mean plots to accommodate winter maxima of warming over high-latitude northern hemisphere land.

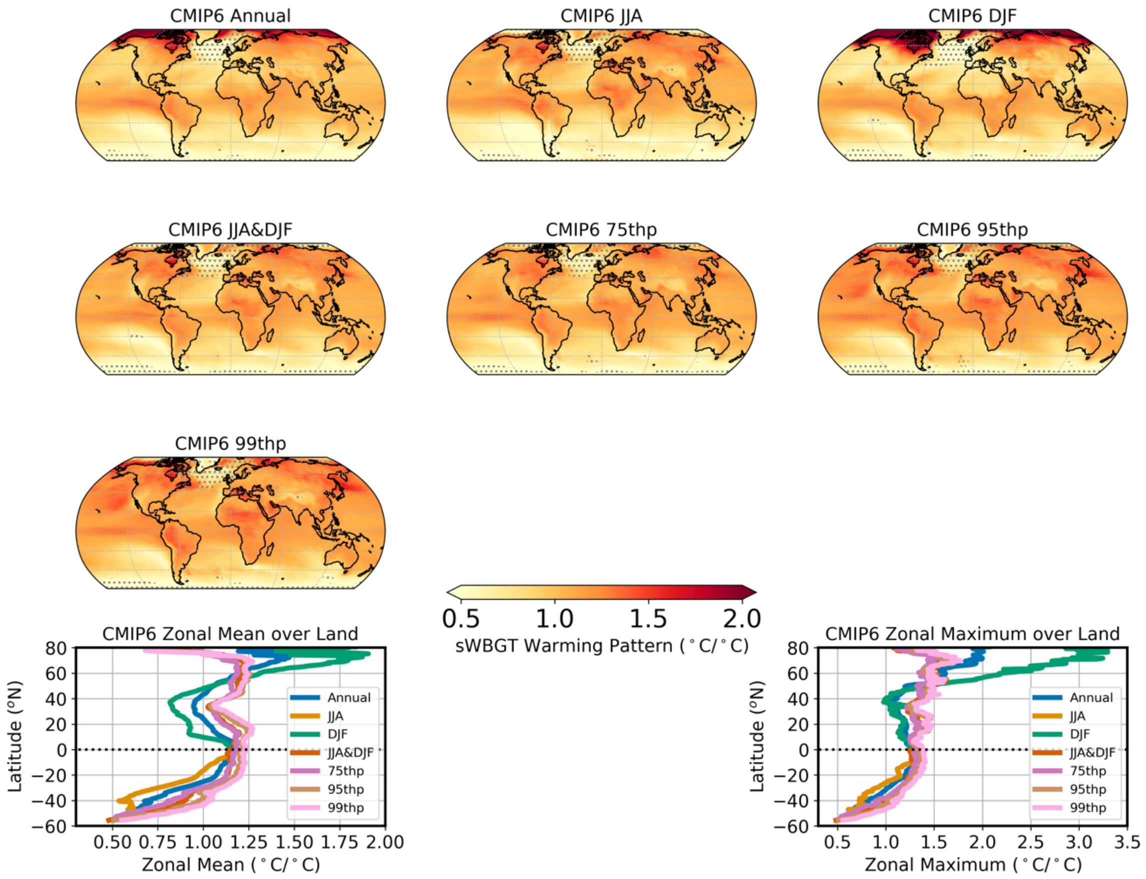

Supplementary Figure 6. Maps showing multi-model median simplified Wet Bulb Globe Temperature (sWBGT) annual mean, JJA, DJF, JJA/DJF (concatenated at equator, JJA for northern Hemisphere, DJF for southern Hemisphere), 75th percentile, 95th percentile, and 99th percentile warming patterns per degree of global warming in 21 CMIP6 1%CO2 simulations. Line plots show zonal mean (lower left) and zonal maximum (bottom right) sWBGT warming patterns over land. Stippling on maps shows where models disagree on the magnitude of local warming per degree of global warming (Methods). Note the different x-axis limits in the zonal mean plots to accommodate winter maxima of warming over high-latitude northern hemisphere land.

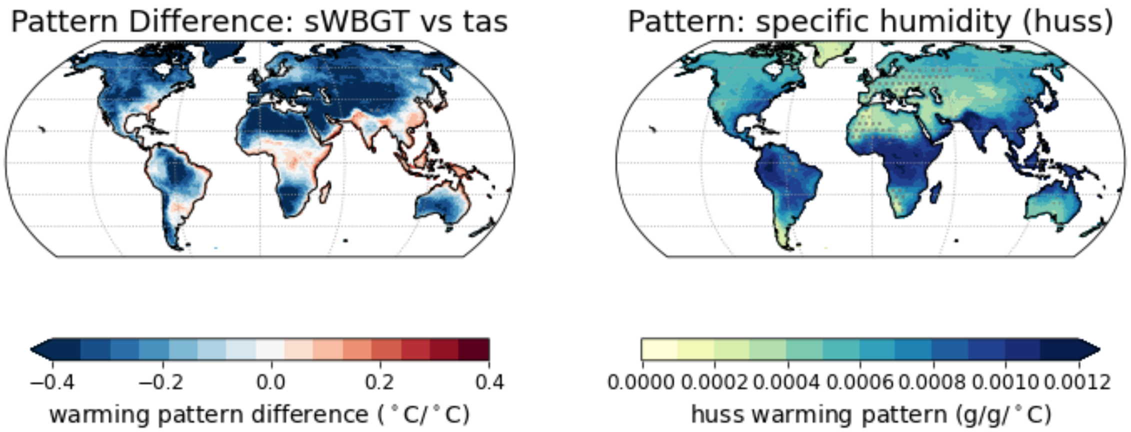

Supplementary Figure 7. Map showing the CMIP6 multi-model median difference in warming patterns among sWBGT and 2-m air temperature (left) and specific humidity warming pattern (right). Note that regions showing faster local increases in sWBGT relative to temperature increases (red shading) tend to be co-located with regions of rapid increases in specific humidity per degree of global warming. Region showing slower increases in sWBGT as compared to changes in local temperatures (blue shading) per degree of global warming tend to be co-located with locations showing minimal specific humidity changes. Stippling shows where <75% of the 21 CMIP6 models agree on the sign of difference in sWBGt-tas warming patterns per degree of global warming, and in the humidity maps where the coefficient of variation is >0.35 (see Methods in main text). The pattern correlation coefficient among the sWBGT-temperature difference and specific humidity warming pattern is r = 0.84.

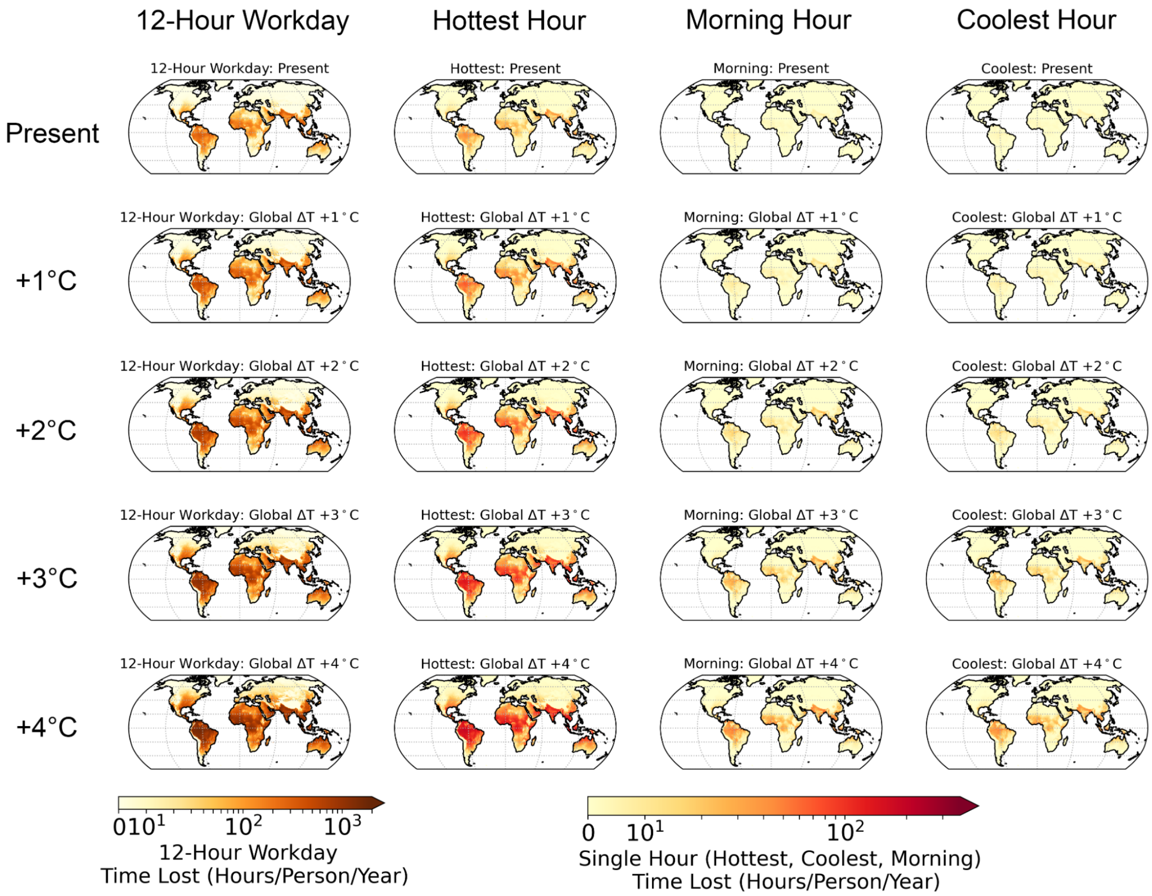

Supplementary Figure 8. Work hours lost for the full 12-hour work day (left column), hottest hour around midday (center left column), early morning hour (center right), and coolest hour of the day (right column) for individuals conducting heavy manual labor for annual-mean, global-mean temperature changes of 0°C (present day: 2001-2020), +1°C, +2°C, +3°C, and +4°C (top to bottom). Simplified Wet Bulb Globe Temperature (sWBGT) warming patterns from Supplementary Figure 3 have been added to the ERA5 sWBGT for each global warming threshold, then the average work time lost is calculated. Units are in hours per year per person. Note the 12-hour workday white-yellow-brown colorbar shows a maximum of 4380 hours/person/year, and the white-yellow-red colorbar shows a maximum of 365 hours/person/year for the midday/morning/sunrise maps.

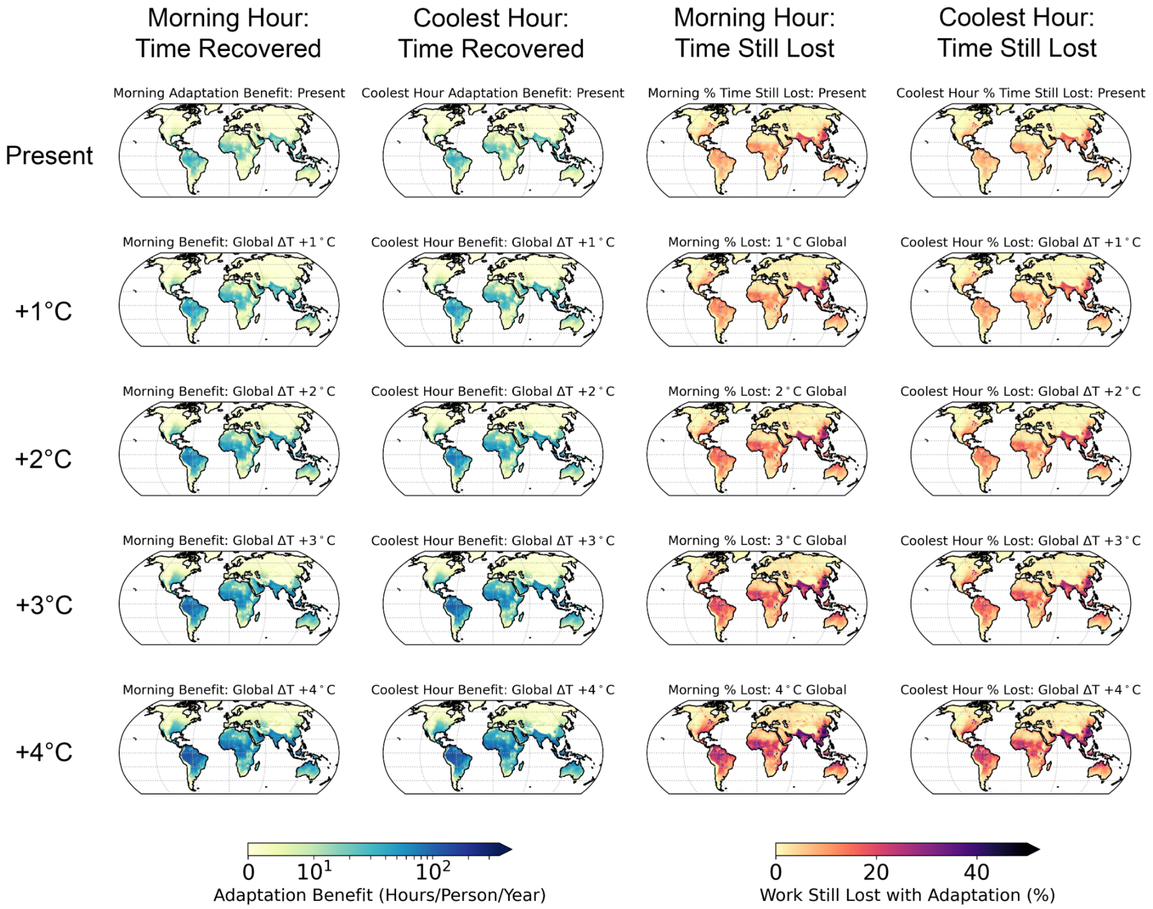

Supplementary Figure 9. Adaptation benefit (left columns) and work time still lost (right columns) via moving work hours from the hottest hour of the day to cooler hours with global warming. Left columns (blue-green colormap) show work hours saved ('adaptation benefit') if workers are able to adapt to warming by moving heavy labor from midday to the morning (left column) or to the coolest hour of the day around sunrise (center left column). Right columns (yellow-purple colormap) show percent of midday lost work time that cannot be regained by moving work hours to the morning (center right column) and to sunrise (right column) due to increasing heat exposure in these morning hours as the planet warms. Results shown for individuals conducting heavy manual labor for annual-mean, global-mean temperature changes of 0°C (present day: 2001-2020), +1°C, +2°C, +3°C, and +4°C. Simplified Wet Bulb Globe Temperature (sWBGT) warming patterns from Supplementary Figure 3 have been added to the ERA5 sWBGT for each global warming threshold, then the average work time lost is calculated. Units in left columns are in hours per year per person. Units in right columns are shown as a percent of time lost in the morning hours relative to time lost in the midday hour.

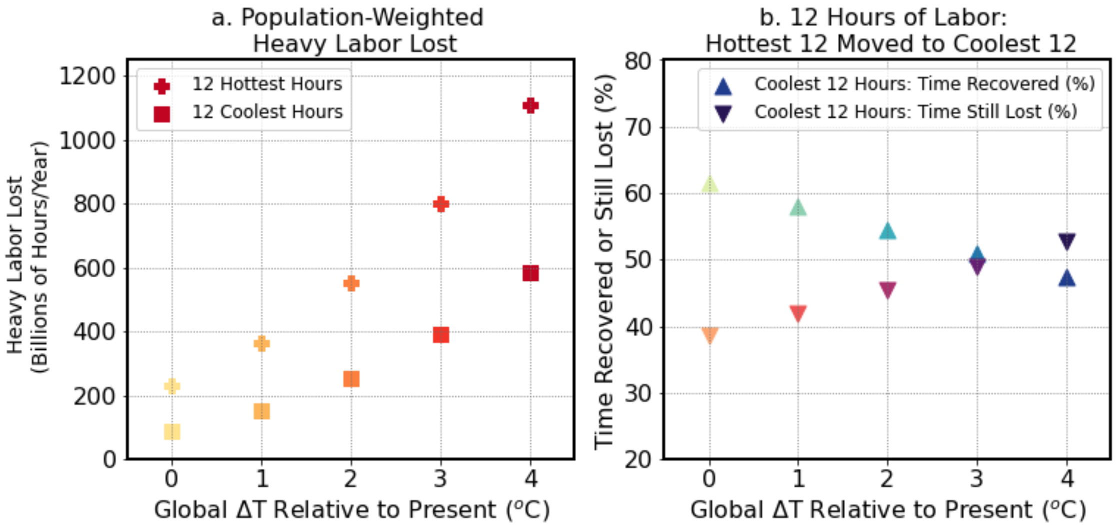

Supplementary Figure 10. Global sums of heavy labor lost in the hottest 12 hours of the day and in the coolest 12 hours of the day (a) for global-mean temperature changes of 0°C (present day: 2001-2020), +1°C, +2°C, +3°C, and +4°C. Percent of time (b) that can be recovered (or is still lost) by moving labor from the hottest 12 hours of the day to the coolest 12 hours of the day. Population-weighted sums are calculated by multiplying gridded per-capita losses by gridded working-age population in each labor sector.

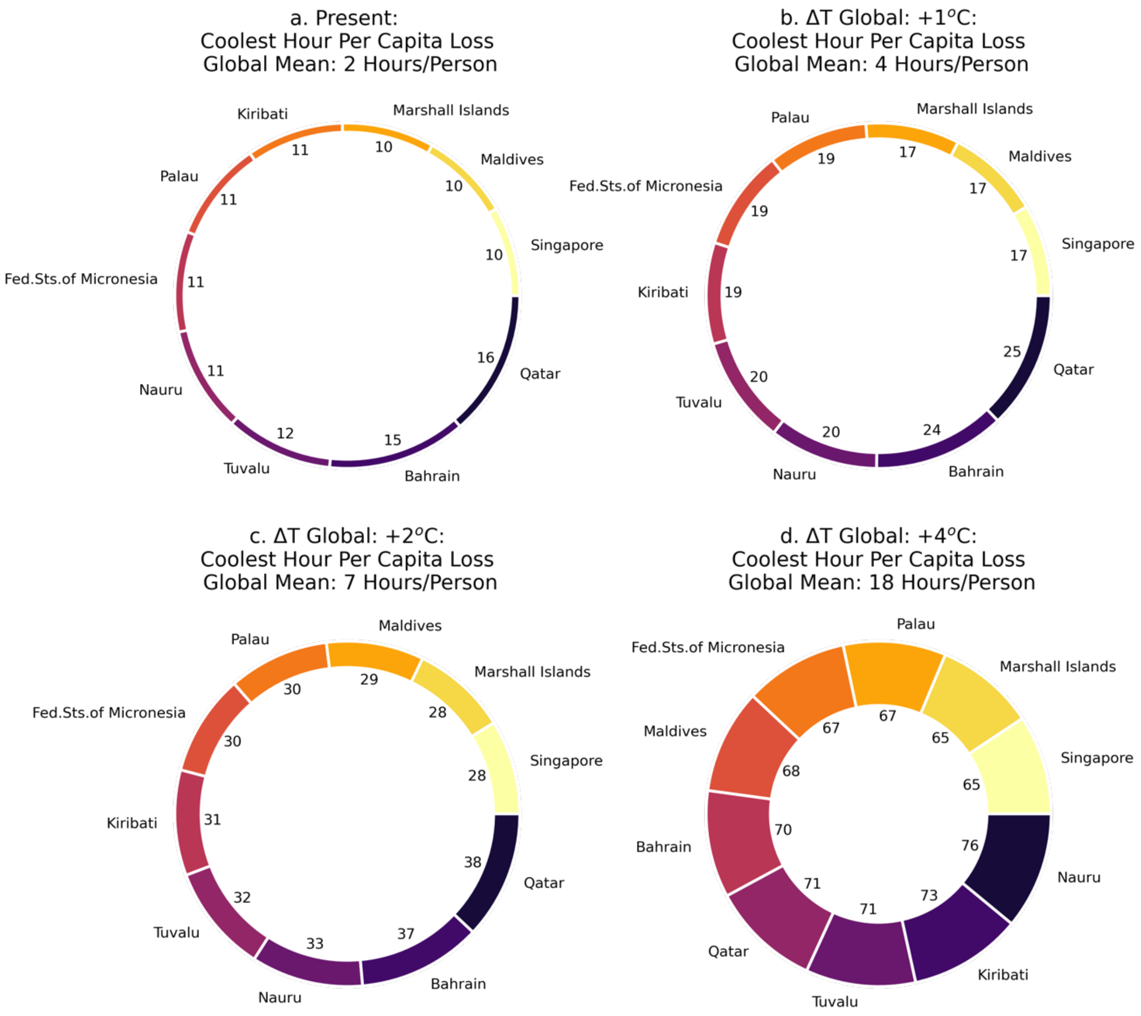

Supplementary Figure 11. The 10 countries with the most losses in heavy labor due to heat exposure at the coolest hour of the day in the present (2001-2020 mean), and with +1°C, +2°C, and +4°C of additional warming. The global mean (average of 163 countries; Table S2) of labor lost is shown above each plot, the numbers around the center of the circle show individual losses per country, and the thickness of the donut plots increase as the global mean of labor lost increases with warming. Units are in hours/person/year.

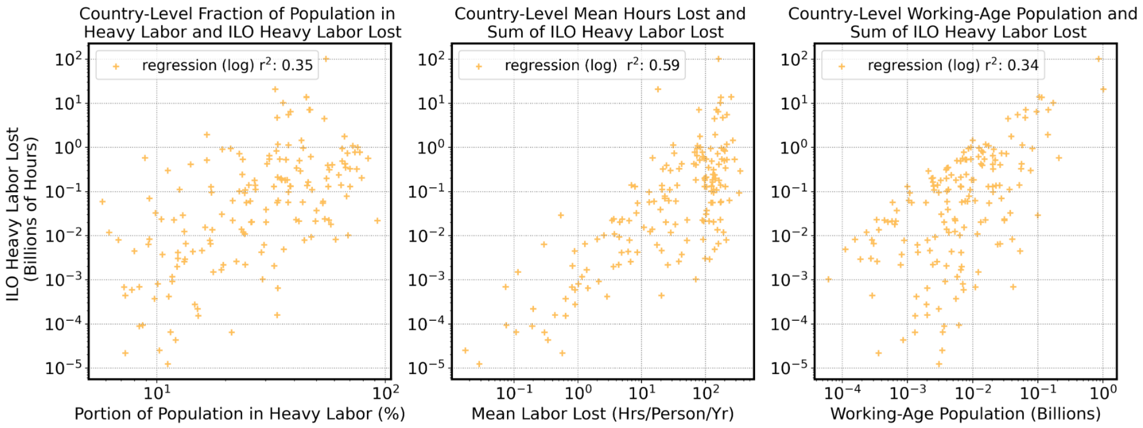

Supplementary Figure 12. Scatter plots showing the country-level relationships among the portion of the population working in heavy labor (left), per capita labo lost (center), and working-age population (right) plotted against country-level, population-weighted ILO heavy labor losses in the recent past (2001-2020 mean labor losses). Note axes are shown on a log scale. Plot legends show the variance explained (r-squared statistic from regression) when the in log-transformed regression calculations.

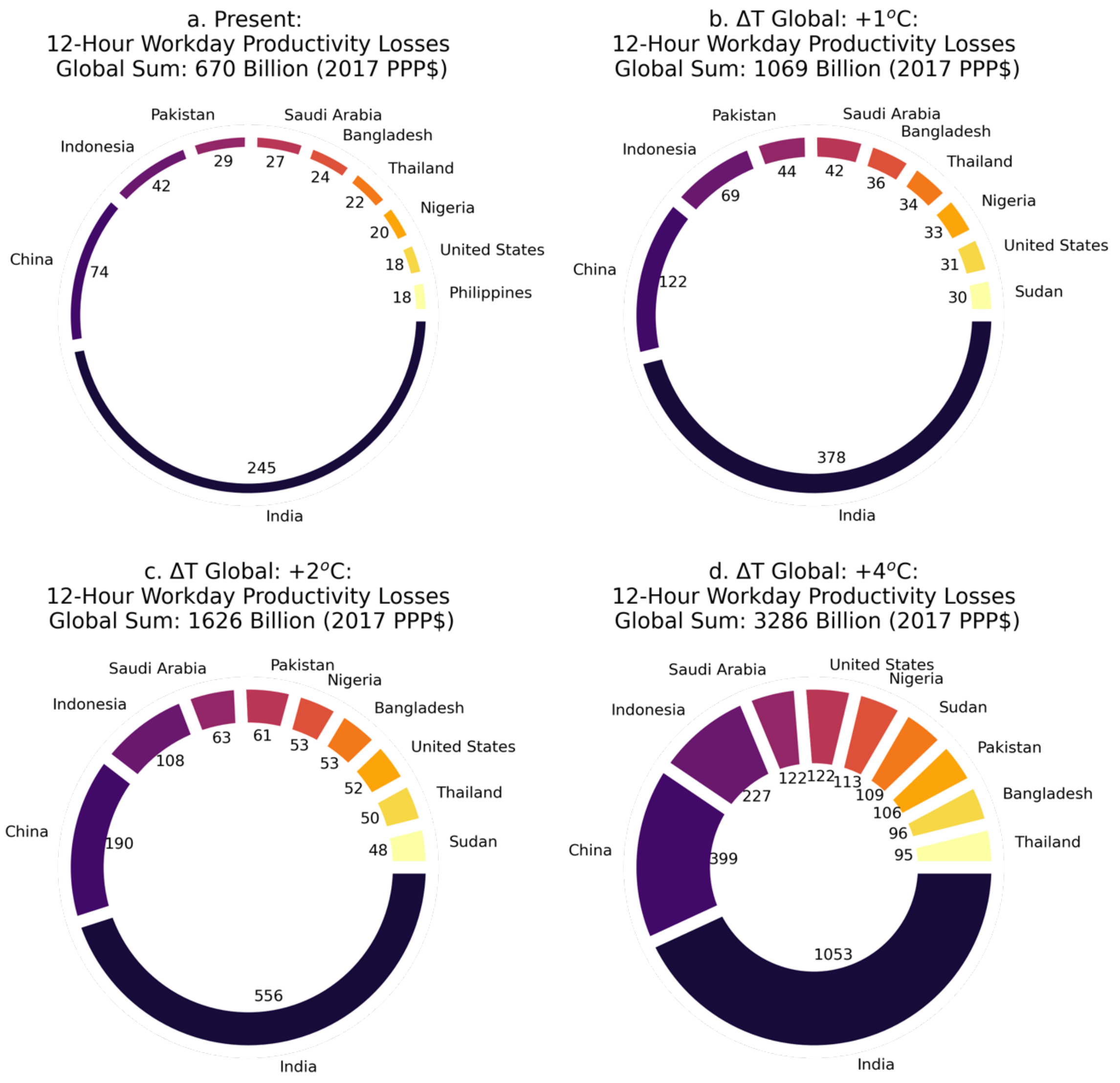

Supplementary Figure 13. The 10 countries with the most economic productivity (2017 PPP$) losses in the 12-hour workday in heavy labor due to heat exposure in the present (a, 2001-2020 mean), and with +1°C, +2°C, and +4°C of additional warming (b-d). The global mean of productivity lost (average of 163 countries with available data; Supplementary Table 1) is shown above each plot, the numbers around the center of the circle show individual losses per country, and the thickness of the circle increases as the global mean of productivity lost increases with warming. All units are in hours/person/year.

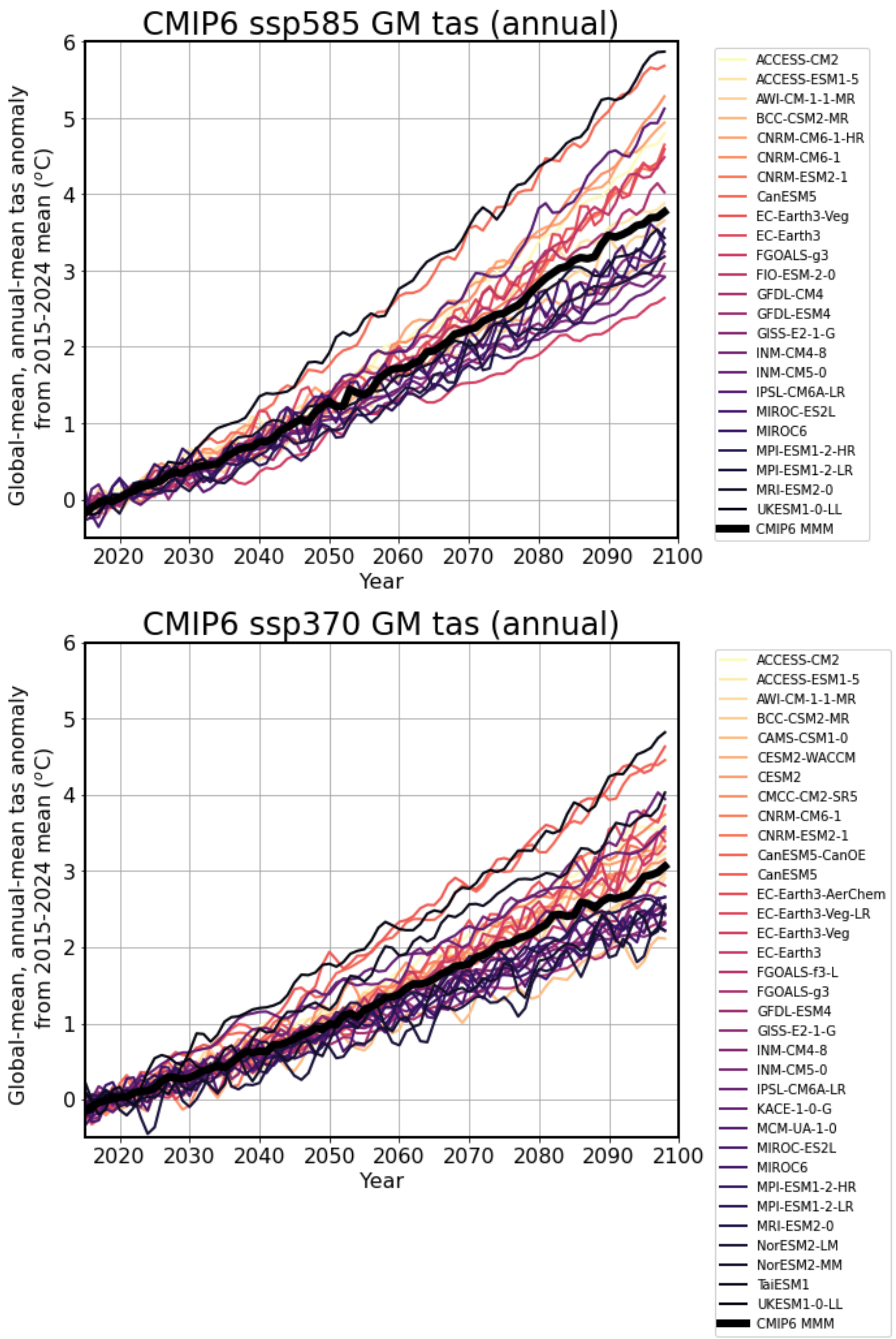

Supplementary Figure 14. Time series showing 21st century annual, global-mean, latitude-area weighted 2-m air temperature anomalies from the 2015-2024 mean from the CMIP6 SSP5-8.5 (top) and SSP3-7.0 (bottom) experiments. Colored lines show individual model results, and the thick black line shows the multi-model median (CMIP6 MMM).

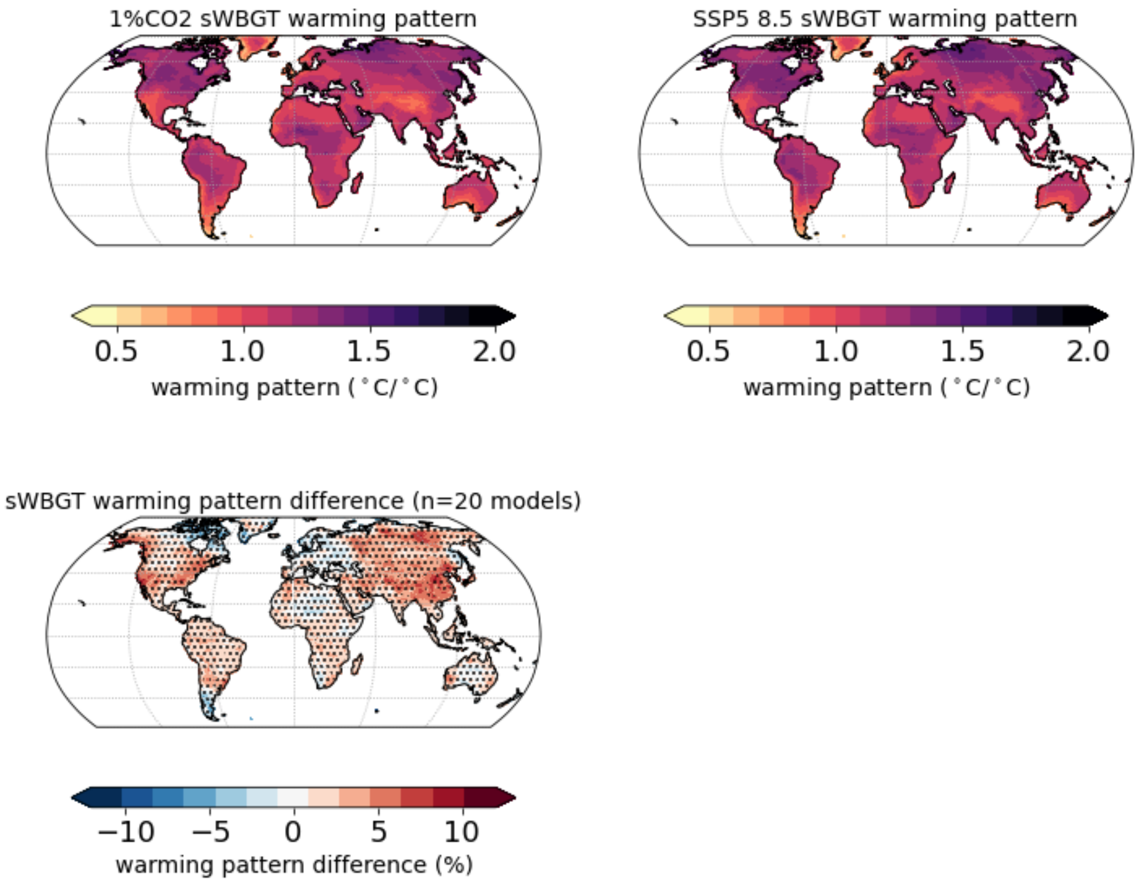

Supplementary Figure 15. Warm-season local sWBGT warming patterns per degree of annual-mean, global mean temperature change in 20 1%CO2 and 20 SSP58.5 CMIP6 simulations (yellow-maroon colormap) and warming pattern differences (blue-white-red colormap). On the difference map, red shading indicates higher-magnitude local change in the SSP58.5 experiment, and blue shading indicates higher-magnitude local change in the 1%CO2 experiment. Stippling shows where <75% of CMIP6 models agree on the sign of difference in warming patterns among the experiments. Note that models agree on the sign of change over several mid-latitude northern hemisphere regions, but these differences are generally <10% relative to the magnitude of change in the 1%CO2 multi-model median. All necessary variables were not available to calculate sWBGT in the SSP58.5 experiment for the CMIP6 'MIROC6' and 'SAM0-UNICON' models so maps show multi-model medians and differences for 19 of the 21 models shown in Figure 2.

Supplementary Table 1. List of countries (n=163), working-age population in 2017 from the GPWv4 data, percent of the working-age population in agriculture+forestry+fisheries and construction, percent in agriculture+forestry+fisheries alone, and in construction alone.

Country	Working Age (15+) Population in Millions	ILO Ag+Const (%)	ILO Ag (%)	ILO Const (%)
Afghanistan	18.0	53.3	43.4	9.9
Albania	2.0	44.3	37.3	7.0
Algeria	26.1	27.0	10.0	17.0
Angola	14.1	56.0	50.5	5.5
Argentina	27.8	9.3	0.1	9.2
Armenia	2.0	33.9	30.4	3.5
Australia	15.8	11.9	2.6	9.3
Austria	5.8	11.7	3.7	8.0
Azerbaijan	6.9	43.6	36.3	7.3
Bahamas	0.3	12.4	2.2	10.2
Bahrain	1.1	24.3	1.0	23.3
Bangladesh	105.8	45.2	39.5	5.7
Barbados	0.2	13.3	2.7	10.6
Belarus	6.6	19.2	11.3	7.9
Belgium	7.3	7.8	1.0	6.8
Belize	0.2	23.9	17.1	6.8
Benin	5.7	42.2	39.3	2.9
Bhutan	0.6	57.9	56.0	1.9
Bolivia	6.6	38.6	31.1	7.5
Bosnia and Herzegovina	2.5	24.4	15.7	8.7
Botswana	1.4	29.5	21.0	8.5
Brazil	143.2	16.6	9.3	7.3
Brunei Darussalam	0.3	6.8	1.4	5.4
Bulgaria	4.8	14.4	6.6	7.8
Burkina Faso	9.4	31.1	25.9	5.2
Burundi	5.4	92.5	92.0	0.5
Cambodia	10.0	43.0	33.7	9.3
Cameroon	12.3	47.2	44.1	3.1
Canada	24.4	9.2	1.5	7.7
Cape Verde	0.3	22.3	11.8	10.5
Central African Republic	2.5	78.9	77.6	1.3
Chad	7.4	77.5	76.7	0.8
Chile	12.2	17.7	9.2	8.5
China	1013.5	33.0	26.1	6.9

**Colombia**	33.1	23.0	16.7	6.3
**Comoros**	0.4	56.8	50.6	6.2
**Congo**	2.5	38.4	34.6	3.8
**Congo Democratic Republic of the**	39.1	66.4	65.8	0.6
**Costa Rica**	3.3	19.3	12.4	6.9
**Croatia**	2.8	13.2	6.2	7.0
**Cuba**	8.0	23.7	17.8	5.9
**Cyprus**	0.8	10.6	2.2	8.4
**Czechia**	7.1	10.1	2.8	7.3
**Cote dIvoire**	12.6	43.8	40.9	2.9
**Denmark**	3.7	8.4	2.2	6.2
**Djibouti**	0.6	37.6	33.9	3.7
**Dominican Republic**	6.7	17.3	9.3	8.0
**Ecuador**	10.4	35.3	28.8	6.5
**Egypt**	57.9	37.7	24.4	13.3
**El Salvador**	4.0	23.0	16.7	6.3
**Equatorial Guinea**	0.7	50.0	42.1	7.9
**Eritrea**	2.9	64.0	61.6	2.4
**Estonia**	0.9	12.1	3.3	8.8
**Eswatini**	0.8	19.6	12.7	6.9
**Ethiopia**	55.3	70.2	66.7	3.5
**Fiji**	0.6	42.0	36.9	5.1
**Finland**	3.5	11.5	3.7	7.8
**France**	40.5	9.3	2.5	6.8
**Gabon**	1.2	33.7	33.3	0.4
**Gambia**	1.0	34.0	27.8	6.2
**Georgia**	2.6	47.5	42.4	5.1
**Germany**	53.7	8.0	1.3	6.7
**Ghana**	15.9	33.9	30.4	3.5
**Greece**	7.4	16.3	12.3	4.0
**Guatemala**	9.7	37.3	31.7	5.6
**Guinea**	6.6	64.8	62.3	2.5
**Guinea-Bissau**	1.0	70.2	68.5	1.7
**Guyana**	0.5	26.1	17.5	8.6
**Haiti**	6.6	33.8	29.0	4.8
**Honduras**	5.6	36.5	30.7	5.8
**Hungary**	6.7	12.4	4.9	7.5
**Iceland**	0.2	10.3	4.0	6.3
**India**	859.9	55.1	43.3	11.8
**Indonesia**	173.1	35.7	29.6	6.1

**Iran Islamic Republic of**	56.5	30.4	17.7	12.7
**Iraq**	20.4	31.1	18.4	12.7
**Ireland**	3.1	11.2	4.8	6.4
**Israel**	4.3	6.2	1.0	5.2
**Italy**	38.0	9.9	3.8	6.1
**Jamaica**	1.9	24.7	16.2	8.5
**Japan**	78.1	11.2	3.5	7.7
**Jordan**	5.4	13.2	3.1	10.1
**Kazakhstan**	11.8	24.3	16.3	8.0
**Kenya**	26.4	58.6	55.1	3.5
**Korea Democratic People's Republic of**	17.5	56.3	51.5	4.8
**Korea Republic of**	37.0	12.6	5.0	7.6
**Kuwait**	3.0	17.4	2.1	15.3
**Kyrgyzstan**	4.0	32.7	22.0	10.7
**Lao People's Democratic Republic**	4.2	67.2	63.2	4.0
**Latvia**	1.3	15.2	7.0	8.2
**Lebanon**	4.0	23.5	13.8	9.7
**Lesotho**	1.3	21.3	8.8	12.5
**Liberia**	2.5	46.0	43.4	2.6
**Libya**	4.2	30.1	18.4	11.7
**Lithuania**	2.0	14.7	7.2	7.5
**Luxembourg**	0.4	7.3	1.1	6.2
**Madagascar**	13.5	66.6	64.7	1.9
**Malawi**	9.2	47.9	44.1	3.8
**Malaysia**	21.2	19.4	10.7	8.7
**Maldives**	0.3	13.4	8.7	4.7
**Mali**	8.7	64.8	63.0	1.8
**Malta**	0.3	7.3	1.0	6.3
**Mauritania**	2.3	55.4	52.1	3.3
**Mauritius**	0.9	17.1	6.3	10.8
**Mexico**	83.1	21.1	12.8	8.3
**Mongolia**	2.0	33.7	28.0	5.7
**Morocco**	23.0	45.5	35.3	10.2
**Mozambique**	14.5	73.7	70.6	3.1
**Myanmar**	35.1	54.1	49.7	4.4
**Namibia**	1.4	28.7	22.6	6.1
**Nepal**	17.6	72.1	65.6	6.5
**Netherlands**	11.1	7.2	2.1	5.1
**New Zealand**	3.0	15.1	5.8	9.3

**Nicaragua**	4.0	35.0	30.3	4.7
**Niger**	9.4	75.9	75.3	0.6
**Nigeria**	96.2	38.5	35.6	2.9
**Norway**	3.4	10.3	2.1	8.2
**Oman**	3.2	26.8	4.7	22.1
**Pakistan**	114.7	45.2	37.4	7.8
**Panama**	2.6	24.4	14.2	10.2
**Papua New Guinea**	4.7	62.6	59.4	3.2
**Paraguay**	4.2	27.1	20.1	7.0
**Peru**	20.5	32.6	27.4	5.2
**Philippines**	64.3	33.7	24.3	9.4
**Poland**	26.6	17.2	9.6	7.6
**Portugal**	6.8	12.3	6.0	6.3
**Qatar**	2.1	42.6	1.2	41.4
**Romania**	13.4	30.1	22.3	7.8
**Russian Federation**	100.2	13.0	5.9	7.1
**Rwanda**	6.4	68.8	63.3	5.5
**Samoa**	0.1	36.0	30.8	5.2
**Saudi Arabia**	22.3	16.6	2.5	14.1
**Senegal**	8.1	36.8	30.7	6.1
**Sierra Leone**	3.9	56.9	55.6	1.3
**Singapore**	4.1	5.8	0.7	5.1
**Slovakia**	3.8	11.7	2.3	9.4
**Slovenia**	1.4	11.3	5.5	5.8
**Solomon Islands**	0.3	40.7	38.1	2.6
**Somalia**	7.0	84.2	83.2	1.0
**Spain**	30.7	10.5	4.2	6.3
**Sri Lanka**	13.7	33.4	25.2	8.2
**Sudan**	145.7	46.9	40.1	6.8
**Suriname**	0.4	17.5	7.6	9.9
**Sweden**	6.2	8.7	1.7	7.0
**Switzerland**	5.5	9.8	3.0	6.8
**Syrian Arab Republic**	10.9	21.5	11.0	10.5
**Tajikistan**	5.3	54.4	45.8	8.6
**Tanzania United Republic of**	27.9	68.3	65.8	2.5
**Thailand**	49.0	37.7	32.1	5.6
**Timor-Leste**	0.7	49.5	45.5	4.0
**Togo**	4.0	40.9	38.3	2.6
**Tonga**	0.1	29.1	24.5	4.6
**Tunisia**	7.8	25.7	13.3	12.4

**Turkey**	52.1	25.3	18.4	6.9
**Turkmenistan**	3.6	25.9	20.3	5.6
**Uganda**	20.7	75.1	72.9	2.2
**Ukraine**	30.9	21.5	14.9	6.6
**United Arab Emirates**	7.7	23.8	1.5	22.3
**United Kingdom**	42.0	8.4	1.1	7.3
**United States**	211.7	8.9	1.4	7.5
**Uruguay**	2.2	15.8	8.4	7.4
**Uzbekistan**	20.7	37.7	24.6	13.1
**Vanuatu**	0.2	60.6	56.4	4.2
**Venezuela Bolivarian Republic of**	20.4	14.1	8.0	6.1
**Viet Nam**	65.7	46.6	38.6	8.0
**Yemen**	15.3	34.6	29.4	5.2
**Zambia**	8.4	53.5	49.3	4.2
**Zimbabwe**	8.8	67.9	66.3	1.6

Supplementary Table 2. List of CMIP6 models and ensemble members used to generate sWBGT warming patterns used in the main text.

CMIP6 Model	Experiment	Ensemble Member
ACCESS-CM2	1pctCO2, SSP5 8.5	r1i1p1f1
ACCESS-ESM1-5	1pctCO2, SSP5 8.5	r1i1p1f1
CanESM5	1pctCO2, SSP5 8.5	r1i1p1f1
CESM2	1pctCO2, SSP5 8.5	r1i1p1f1
CNRM-CM6-1	1pctCO2, SSP5 8.5	r1i1p1f2
CNRM-CM6-1-HR	1pctCO2, SSP5 8.5	r1i1p1f2
CNRM-ESM2-1	1pctCO2, SSP5 8.5	r1i1p1f2
FGOALS-g3	1pctCO2, SSP5 8.5	r1i1p1f1
GFDL-CM4	1pctCO2, SSP5 8.5	r1i1p1f1
GFDL-ESM4	1pctCO2, SSP5 8.5	r1i1p1f1
HadGEM3-GC31-LL	1pctCO2, SSP5 8.5	r1i1p1f3
HadGEM3-GC31-MM	1pctCO2, SSP5 8.5	r1i1p1f3
INM-CM4-8	1pctCO2, SSP5 8.5	r1i1p1f1
KIOST-ESM	1pctCO2, SSP5 8.5	r1i1p1f1
MIROC6	1pctCO2	r1i1p1f1
MIROC-ES2L	1pctCO2, SSP5 8.5	r1i1p1f2
MRI-ESM2-0	1pctCO2, SSP5 8.5	r1i1p1f1
NorESM2-LM	1pctCO2, SSP5 8.5	r1i1p1f1
NorESM2-MM	1pctCO2, SSP5 8.5	r1i1p1f1
SAM0-UNICON	1pctCO2	r1i1p1f1
UKESM1-0-LL	1pctCO2, SSP5 8.5	r1i1p1f2

Supplementary References

1 Wang, K. & Clow, G. D. The Diurnal Temperature Range in CMIP6 Models: Climatology, Variability, and Evolution. *Journal of Climate* **33**, 8261-8279 (2020).

2 Thorne, P. W. *et al.* Reassessing changes in diurnal temperature range: Intercomparison and evaluation of existing global data set estimates. *Journal of Geophysical Research: Atmospheres* **121**, 5138-5158 (2016).

3 Lindvall, J. & Svensson, G. The diurnal temperature range in the CMIP5 models. *Climate Dynamics* **44**, 405-421 (2015).

4 Di Luca, A., de Elia, R., Bador, M. & Argüeso, D. Contribution of mean climate to hot temperature extremes for present and future climates. *Weather and Climate Extremes* **28**, 100255 (2020).

5 Buzan, J. R. & Huber, M. Moist heat stress on a hotter Earth. *Annual Review of Earth and Planetary Sciences* **48**, 623-655 (2020).

6 Kjellstrom, T., Freyberg, C., Lemke, B., Otto, M. & Briggs, D. Estimating population heat exposure and impacts on working people in conjunction with climate change. *International journal of biometeorology* **62**, 291-306 (2018).

7 Watts, N. *et al.* The 2020 report of The Lancet Countdown on health and climate change: responding to converging crises. *The Lancet* (2020).
