# Supplementary material for: Increased labor losses and decreased adaptation potential in a warmer world
Source: Nat Commun. 2021 Dec 14;12:7286. doi: 10.1038/s41467-021-27328-y (PMC8671389; doi:10.1038/s41467-021-27328-y)
Supplement: Supplementary file 4 — Reporting Summary [file 41467_2021_27328_MOESM4_ESM.pdf]

nature portfolio

Corresponding author(s): Luke Parsons

Last updated by author(s): Oct 25, 2021

Reporting Summary

Nature Portfolio wishes to improve the reproducibility of the work that we publish. This form provides structure for consistency and transparency in reporting. For further information on Nature Portfolio policies, see our Editorial Policies and the Editorial Policy Checklist.

Statistics

For all statistical analyses, confirm that the following items are present in the figure legend, table legend, main text, or Methods section.

n/a	Confirmed	
[ ]	[x]	The exact sample size (n) for each experimental group/condition, given as a discrete number and unit of measurement
[x]	[ ]	A statement on whether measurements were taken from distinct samples or whether the same sample was measured repeatedly
[x]	[ ]	The statistical test(s) used AND whether they are one- or two-sided<br>*Only common tests should be described solely by name; describe more complex techniques in the Methods section.*
[x]	[ ]	A description of all covariates tested
[x]	[ ]	A description of any assumptions or corrections, such as tests of normality and adjustment for multiple comparisons
[ ]	[x]	A full description of the statistical parameters including central tendency (e.g. means) or other basic estimates (e.g. regression coefficient) AND variation (e.g. standard deviation) or associated estimates of uncertainty (e.g. confidence intervals)
[x]	[ ]	For null hypothesis testing, the test statistic (e.g. F, t, r) with confidence intervals, effect sizes, degrees of freedom and P value noted<br>*Give P values as exact values whenever suitable.*
[x]	[ ]	For Bayesian analysis, information on the choice of priors and Markov chain Monte Carlo settings
[x]	[ ]	For hierarchical and complex designs, identification of the appropriate level for tests and full reporting of outcomes
[x]	[ ]	Estimates of effect sizes (e.g. Cohen's d, Pearson's r), indicating how they were calculated

Our web collection on statistics for biologists contains articles on many of the points above.

Software and code

Policy information about availability of computer code

Data collection	All data were downloaded from free, publicly available data repositories. No code was necessary to download the data, but the ECMWF data portal does provide a Python-based API.
Data analysis	Python code provided by Li et al. to calculate hourly sWBGT from ERA5 data are available on GitHub (https://github.com/dw-li/WBGT). Python code were provided by Chavaillaz et al. to calculate WBGT from CMIP data. Code is available from Li et al. and Chavaillaz et al., or the corresponding author upon reasonable request. Code used to plot diurnal cycles of sWBGT, CMIP6 monthly warming patterns, and global sums of labor losses are available on GitHub: https://github.com/LukeAParsons/Warming_Adaptation_Limits.

For manuscripts utilizing custom algorithms or software that are central to the research but not yet described in published literature, software must be made available to editors and reviewers. We strongly encourage code deposition in a community repository (e.g. GitHub). See the Nature Portfolio guidelines for submitting code & software for further information.

Data

Policy information about availability of data

All manuscripts must include a data availability statement. This statement should provide the following information, where applicable:

- Accession codes, unique identifiers, or web links for publicly available datasets
- A description of any restrictions on data availability
- For clinical datasets or third party data, please ensure that the statement adheres to our policy

CMIP6 experimental output ('orog' and daily data variables: 'tas', 'tasmax', 'tasmean', 'huss', 'psl') can be found at: https://esgf-node.llnl.gov/search/cmip6/.
Hourly single level reanalysis ERA5 data can be found at https://cds.climate.copernicus.eu/#!/search?text=ERA5&type=dataset.
ILO sector-specific labor and earnings data can be found on the ILOSTAT data explorer: https://www.ilo.org/shinyapps/bulkexplorer7/?

lang=en&segment=indicator&id=SDG_0111_SEX_AGE_RT_A.
World Bank data can be downloaded from: https://data.worldbank.org/.
GPW v4 population data are available at: https://sedac.ciesin.columbia.edu/data/collection/gpw-v4
Average diurnal cycles of sWBGT, gridded CMIP6 monthly warming patterns, gridded heavy labor productivity losses, and global sums of heavy labor losses calculated from sWBGT data are available on Zenodo: 10.5281/zenodo.5594470.

Field-specific reporting

Please select the one below that is the best fit for your research. If you are not sure, read the appropriate sections before making your selection.

☐ Life sciences ☐ Behavioural & social sciences ☒ Ecological, evolutionary & environmental sciences

For a reference copy of the document with all sections, see nature.com/documents/nr-reporting-summary-flat.pdf

Ecological, evolutionary & environmental sciences study design

All studies must disclose on these points even when the disclosure is negative.

Study description	We used hourly ERA5 reanalysis data to calculate the humid heat metric sWBGT. We applied the previously published (Kjellstrom et al., 2018; Watts et al., 2021) exposure response function that relates WBGT to labor productivity to the hourly sWBGT data (1979-2020 and 2001-2020) to calculate annual labor productivity losses (and mean losses 2001-2020) due to humid heat exposure. We compared historical losses in the hottest hours of the day to losses in the coolest hours of the day. We then calulated sWBGT in the CMIP6 data, and used warming patterns from the CMIP6 models to add 1, 2, 3, and 4C of warming to the hourly (2001-2020) ERA5 data to project potential future warming impacts on labor productivity losses if the diurnal cycle was warmed at each of these warming levels. We overlaid ILO statistics on country-level working sectors (agriculture, construction) on spatially gridded (GPWv4) population data to estimate the number of workers and hours lost due to humid heat exposure. We then overlaid country-level World Bank estimates of sector-level contributions (agriculture, industry) to each country's GDP to estimate how these work productivity losses would impact the economy of each country. Data were summed over all countries with matching data across the ILO and World Bank datasets (n=163) to estimate global labor loss and economic productivity loss impacts.
Research sample	CMIP6 data: daily 'tasmax', 'tasmin', 'tas', 'huss', 'psl' variables from the 1pctCO2 (time range: years 1-150) and ssp585 (time range: years 2015-2100) experiments and the time invariant orography ('orog') variable were downloaded from the Earth System Grid Federation data portal.<br>ERA5 data: hourly 't2m', 'd2m', and 'sp' data were downloaded from the ECMWF data portal (time range: Jan 1 1979 - Dec 31 2020).
Sampling strategy	Sample sizes were: all 21 CMIP6 models (n=21 models). All grid points were used in calculation of warming patterns in the main text, so no data were excluded.<br>For ERA5 data, population-weighted hours lost at each grid point (721x1440) were summed over the entire globe to estimate annual global sums of labor loss due to humid heat exposure. We provide an estimate of mean labor losses in the main text, and use the standard deviation across all years (n=20, 2001-2020) to provide the reader with the interannual variability about the mean. No statistical methods were used to determine sample size. Sample size was based on all available data (n=20 years).<br>For Figure 3, we regress global-mean air temperatures against annual, global sums of labor losses for each year between 1979 and 2020 (n=42 years) to illustrate how global labor losses due to humid heat exposure changes as the globe warms. Again, all available data (years) were used, so no sample size calculate was performed. Sample size was all available years (n=42).
Data collection	Data were downloaded and analyzed by the lead author, L Parsons. No instruments were used to collect the data- all data are from publicly available gridded datasets.
Timing and spatial scale	There is no gap in the ERA5 data or the CMIP6 data.<br>ERA5 data span Jan 1, 1979- Dec 31, 2020 at hourly timesteps. All available data and timesteps were used between 1979 and 2020. The ERA5 reanalysis assimilates observation based data, so data collection stopped in 2020 because a full year of observations is not available for the year 2021. Inclusion of data from part of 2021 would create a false impression of low hours lost in 2021 due to incomplete data, so this year cannot be included.<br>CMIP6 data span years 1-150 (1pctCO2 experiment) and years 2015-2100 (SSP5-8.5 experiment) at daily timesteps. All available data were included from years with atmospheric CO2 concentrations near present (~400ppm) to the end of model simulation years- this rationale is explained in the Methods.<br>In terms of spatial scale, all data were used at each grid point across the globe (for reanalysis data, the grid box size is 721 (latitude) x 1440 (longitude), or about 30km at the equator). All CMIP6 data were regridded to this grid structure when warming patterns of models were added to the reanalysis-based estimates of sWBGT. For calculate of population-weighted hours lost, sWBGT was regridded to a common 0.5x0.5 degree grid because the population data were provided at this grid spacing.
Data exclusions	As we describe in the Supplementary Text 1, in one map in the Supplement showing tasmax and tasmin, we excluded NorESM2-LM model from our analysis of tasmax and tasmin in the 1%CO2 experiment because these variables showed unrealistically high and low changes over land areas as compared to the higher-resolution NorESM2-MM and the other CMIP6 models. These differences are only discussed in the Supplement- these results are not part of the main analysis/Methods or text (e.g., tasmax and tasmin warming patterns are not used as part of the analysis in which warming patterns are used to examine future labor impacts- only the 'tas' variable was used). We state our reasoning and that we excluded this model in the figure caption and in Supplementary Text 1, so this exclusion is made clear, even though the data are not used in the results shown in the main text.
Reproducibility	N/A- Data were from publicly available geophysical data sets. No data changed/were varying- all analysis to make plots was

	conducted in Python, so it is reproducible.
Randomization	N/A - All data were used from all years and all grid points from pre-published, publicly available geophysical data sets, so randomization was not applicable.
Blinding	N/A - All data were used from all years and all grid points from pre-published, publicly available geophysical data sets, so blinding was not applicable.

Did the study involve field work? ☐ Yes ☒ No

Reporting for specific materials, systems and methods

We require information from authors about some types of materials, experimental systems and methods used in many studies. Here, indicate whether each material, system or method listed is relevant to your study. If you are not sure if a list item applies to your research, read the appropriate section before selecting a response.

Materials & experimental systems

n/a	Involved in the study
☒	☐ Antibodies
☒	☐ Eukaryotic cell lines
☒	☐ Palaeontology and archaeology
☒	☐ Animals and other organisms
☒	☐ Human research participants
☒	☐ Clinical data
☒	☐ Dual use research of concern

Methods

n/a	Involved in the study
☒	☐ ChIP-seq
☒	☐ Flow cytometry
☒	☐ MRI-based neuroimaging
